# Global Vaccinations: New Urgency to Surmount a Triple Threat of Illness, Antiscience, and Anti-Semitism

**DOI:** 10.5041/RMMJ.10491

**Published:** 2023-01-29

**Authors:** Peter J. Hotez

**Affiliations:** 1 Texas Children’s Hospital Center for Vaccine Development, Department of Pediatrics and Molecular Virology and Microbiology, National School of Tropical Medicine, Baylor College of Medicine, Houston, TX, USA; 2 James A. Baker III Institute of Public Policy, Rice University, Houston, TX, USA; 3 Department of Biology, Baylor University, Waco, TX, USA; 4 Hagler Institute of Advanced Study and Scowcroft Institute of International Affairs, Texas A&M University, College Station, TX, USA

**Keywords:** antiscience, vaccines, disinformation

## INTRODUCTION

Because of rising antivaccine activism and some key global policy missteps, we risk eroding more than 70 years of global health gains. This is occurring through an enabled and empowered antiscience ecosystem, with anti-Semitism and the targeting of Jewish biomedical scientists at its core.

## THE DEVELOPMENT OF VACCINES

To understand how this situation evolved or devolved it is helpful to first appreciate the transformative properties of vaccines and vaccinations. As public health interventions no other technology has been more effective. From widespread and global use of a vaccine, the late 1970s saw the eradication of smallpox, and polio neared elimination as a public health problem. During the 1980s, as a young house officer on the Children’s Service at the Massachusetts General Hospital in Boston, I routinely admitted infants and children with *Haemophilus influenzae* type b (Hib) bacterial meningitis, which even after antibiotic treatment often left them with permanent neurologic impairments. At the end of my pediatrics training an improved Hib vaccine was developed by Drs John Robbins (whose family name was changed from Rabinowitz) and Rachel Schneerson (who earned her medical degree in Israel) at the National Institutes of Health and then introduced into pediatric practice; within a few years that disease also largely disappeared.^1^

### Vaccines and the International Community

The power of vaccines extended beyond their direct health benefits and into the sphere of international relations. Albert Sabin was born in Bialystok, Poland, before emigrating to New York with his family and graduating New York University (NYU) medical school in 1931. At the time, NYU was among the few American medical schools enacting liberal policies for admitting Jews. As a pediatrician-scientist heading a vaccine research laboratory at Cincinnati Children’s Hospital in the 1950s, Dr Sabin developed three strains of the poliovirus that could be safely administered by mouth to induce protective immunity. However, his discoveries became an actual vaccine only through back-channel diplomacy between the US State Department and their Soviet counterpart. The arrangement permitted Sabin to bring his poliovirus strains to the USSR, where they were first produced at an industrial scale and administered to millions of Soviet schoolchildren. The oral vaccine now achieving global polio elimination was ultimately developed through vaccine diplomacy between two Cold War foes.^2^

Fueled by such 20th-century successes, beginning in the 2000s the Gates Foundation and international agencies of the United Nations, including the World Bank, UNICEF, and the World Health Organization (WHO), built a new global alliance for vaccines and immunization, known as Gavi. Through Gavi-sponsored activities the number of pediatric lives lost from vaccine-preventable diseases declined dramatically.^3^

## A COUNTERMOVEMENT OF DISINFORMATION

As Elie Wiesel wrote in his 1978 book, *A Jew Today*: “Perhaps Kafka was right: man’s weakness lies not in his inability to obtain victories, but in his inability to make use of them.”^4^ So it was with vaccines and global vaccinations.

Just as Gavi activities began to accelerate, an antivaccine countermovement took shape. It asserted that vaccines caused autism, even though the biomedical scientific community conducted study after study refuting such claims.^5^–^10^ Through the outsized reach of social media, antivaccine activists soon commanded substantial online reach. They gained strength and funds by learning to monetize the web through the sale of phony autism cures, nutritional supplements, and even entire books, which climbed to the top of the Amazon.com booklist in the vaccination category. A first wave of antivaccine groups either expanded or gave way to next- generation actors. For instance, a Washington DC-based watchdog group, the Center for Countering Digital Hate, recently identified 12 empowered or well-financed individuals, the “disinformation dozen,” who (either by themselves or by their organizations) generate much of this antivaccine content. Tragically, first- and second-generation antivaccine groups achieved a slew of gains as multiple pockets of school-entry vaccine refusal and resistance emerged across the United States.^11^

**Figure 1:**
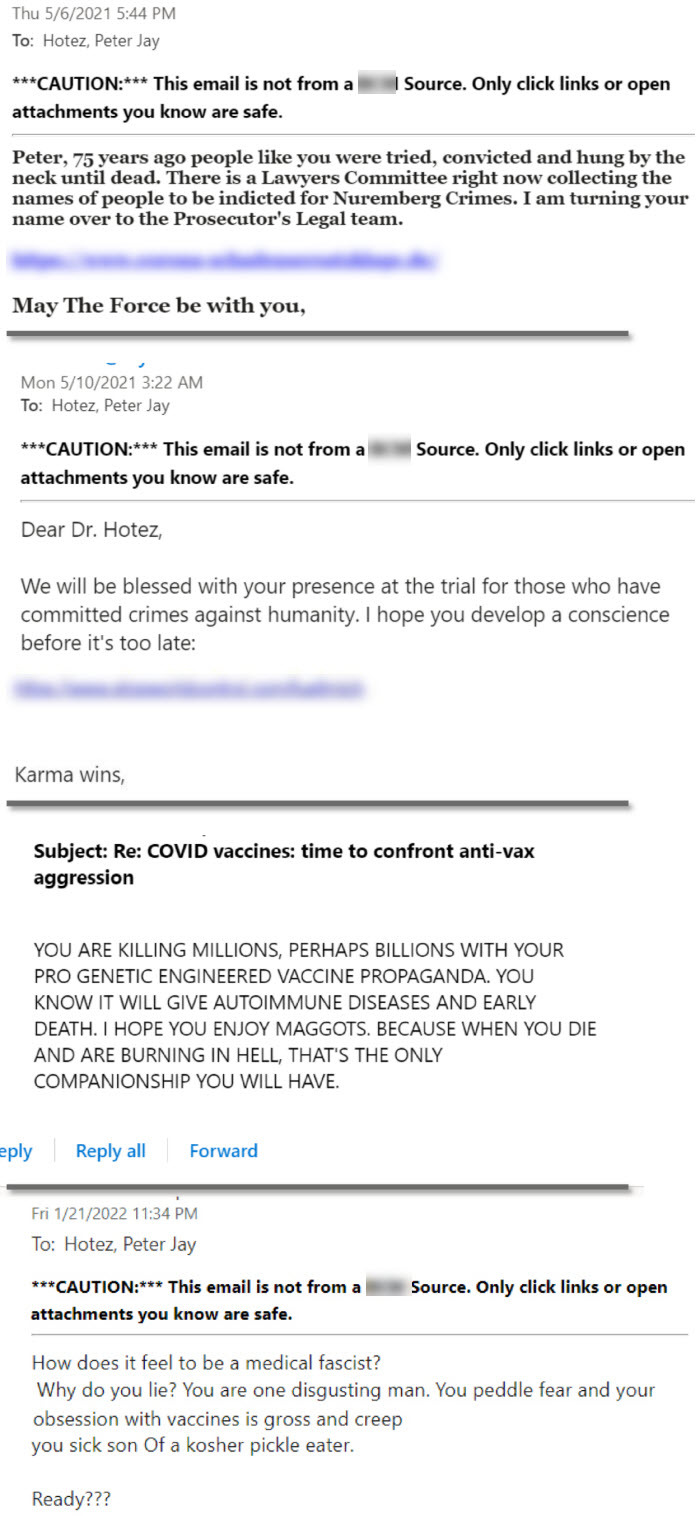
Selected Sample of Personal Emails Received by the Author. Identifying server and sender information removed to protect the identity of the sender.

I am both a vaccine scientist and parent of four adult children, including Rachel who has autism and intellectual disabilities. My book, *Vaccines Did Not Cause Rachel’s Autism*, detailed and summarized the evidence showing conclusively that there is no vaccine–autism link, while offering an alternative narrative: through whole-exome genomic sequencing, Rachel’s autism gene was identified and compared with others like it.^12^ The backlash from antivaccine groups was rapid and severe. They launched a media campaign against me on the internet, encouraging threats through email and social media. Since I make no secret that I am Jewish, I eventually experienced first-hand multiple anti-Semitic statements and threats online (see Figure 1 for examples). Two themes predominated. First there were overt threats or expressions of hatred because I was Jewish. More frequently, however, there were hurtful attempts to accuse me (as well as my colleagues who vaccinate) of perpetrating crimes equivalent to those committed during the Holocaust. Antivaxxers love their Nazi analogies, and I was ultimately compared to the infamous Dr Mengele because I am a scientist who conducts vaccine research, and because I “experimented” on my daughter by ensuring that she still received her recommended vaccinations despite an autism diagnosis. Later emails appearing in my inbox openly expressed their desire to see me hang after some sort of new-age Nuremberg tribunal (Figure 1, top left). I was not alone—a pattern emerged in which Jewish physicians and scientists who conducted vaccine research or advocated for vaccinations were singled out and targeted with Nazi imagery.^13^,^14^

Nevertheless, our scientific community’s efforts to debunk vaccine and autism assertions may have had some beneficial impact, at times even taking some of the wind out of the sails of the antivaccine movement. But this also meant that, to continue, antivaccine groups needed a new angle, and they found it through extremist or libertarian politics. By invoking health freedom or medical freedom propaganda they found a political home and donor support.^15^ Although it may have begun in California with the passage of Senate Bill 277,^15^ which outlawed exemptions to school immunization mandates, health freedom propaganda found its greatest welcome in Texas where an antivaccine political action committee (PAC) formed, and vaccine exemptions escalated.^16^ In time, health freedom and antivaccine sentiments were embraced by many mainstream conservatives or their elected leaders.

The Texas Medical Center where I work is the world’s largest and perhaps most sophisticated collection of biomedical institutions, including Baylor College of Medicine, Texas Children’s Hospital, and the University of Texas MD Anderson Cancer Center, but a dark side in Texas also emerged, the state becoming an epicenter of the antivaccine movement in America. In April 2019 activists demonstrated in front of the Texas State Capitol in Austin, wearing yellow Jewish stars with the words “No Vax” in letters stylized to resemble Hebrew.^17^ The Auschwitz Memorial Anti-Defamation League condemned such protests for mocking the Holocaust and for attempting to intimidate Jewish communities. Despite these condemnations, antivaccine attitudes became entrenched, and activists persuaded parents in Texas to apply for vaccine exemptions in unprecedented numbers. More than 70,000 schoolchildren were denied access to at least one vaccine according to the Texas State Department of Health Services.^18^

In parallel, antivaccine activists targeted insular Orthodox Jewish groups in New York and New Jersey, holding town hall meetings while distributing pamphlets with disinformation. Vaccination rates declined and an outbreak of more than 300 measles cases ensued among these groups in Rockland County and elsewhere in New York in 2018–2019, requiring multiple hospitalizations and intensive care unit (ICU) admissions.^19^ The extent of antivaccine activism among Haredi communities and how much it arose organically versus deliberate targeting by outside forces requires additional study.

## COVID AND THE ANTIVAX MOVEMENT

As the COVID pandemic unfolded, the health freedom language used to oppose childhood immunizations was repurposed to galvanize opposition to COVID vaccines. A Conservative Political Action Coalition (CPAC) Conference held in Dallas in 2021 was especially notable for its open expressions of hostility against COVID vaccines with claims that they were unsafe or ineffective. Extremist members of the US House of Representatives disparaged vaccines, as did several US Senators and Governors. On Twitter one House Member compared vaccinators to “medical brown shirts.”^20^ Tragically, anti-COVID vaccine rhetoric and sentiments were amplified nightly in 2021 by the conservative media. In time, the Proud Boys, a group which according to the Southern Poverty Law Center in the US includes “[R]ank-and file” members and leaders who “regularly spout white nationalist memes and maintain affiliations with known extremists,” began marching at antivaccine results.^21^,^22^

The consequences were devastating: the health analyst, Charles Gaba, as well as multiple news outlets analyzed data to find a sharp partisan political divide in vaccination rates, with overwhelmingly high rates of COVID deaths in Republican-majority states, the so-called “red states,” and counties where COVID vaccination rates were the lowest. *The New York Times* simply labeled it “red COVID.”^23^ Tens of thousands of Americans—overwhelmingly in red states—unnecessarily died in 2021 because they refused a COVID vaccine.^24^

Prominent US scientists espousing the importance of COVID immunizations and other prevention measures also came under threat. After Dr Anthony Fauci, the White House Chief Medical Advisor for COVID-19 and Director of the National Institute of Allergy and Infectious Diseases, I became a second or third favorite target from the extremists in Congress and news outlets. Their public comments became dog whistles for online threats, many imbued with anti-Semitic content and Nazi imagery.^25^–^29^ They blamed the Jews for everything from creating the COVID virus to monetize the pandemic, to selling unsafe or ineffective vaccines.^26^,^27^ I was even stalked at a Houston shul during a high-holiday service. Increasingly, anti-Semitism converged with a growing antiscience ecosystem.

Despite such distractions, it was important to continue our vaccine work. I co-lead a team of scientists at the Texas Medical Center that develops new vaccines for orphaned or neglected diseases. A decade ago, we began developing coronavirus vaccines for SARS and MERS because at that time there was little commercial interest in such vaccines. However, when the COVID virus sequence became available in January 2020 we were able to pivot our program to developing a low-cost COVID vaccine technology.^30^ We did this with no patent, and Baylor licensed it to two vaccine producers, in India and Indonesia, respectively, while we continued to assist them in its industrial production. In India, more than 80 million doses of the vaccine known as CORBEVAX (produced by Biological E) have been distributed to adolescents and adults.^31^ In Indonesia, the vaccine known as INDOVAC (produced by BioFarma) ultimately became one of the few Halal vaccines for Muslim-majority nations. I viewed these efforts as deeply meaningful for both professional and personal reasons. Years before, my cousin and Holocaust survivor, Rabbi Phil Lazowski, of Bloomfield, Connecticut taught me the concept of *tikkun olam—*repairing the world; I committed my life to *vaccine tikkun—*repairing vaccine misinformation, and developing new vaccines for global health.^32^

Nevertheless, the antivaccine groups and political extremists continue to gain strength, and even joining forces to form a type of evil empire that will not stop at COVID vaccinations. Now, they will expand their agenda to target all vaccines and other biomedical interventions. The World Health Organization has already sounded the alarm about declines in childhood vaccinations due to the social disruptions from the pandemic, but a new worry is that global immunization rates will not return to pre-pandemic baselines due to the expansion of antivaccine activism.^33^ In 2022, the first case of paralytic polio in decades occurred in Rockland County, New York. In addition, multiple US biomedical scientists remain under threat.

## COMBATING VACCINE DISINFORMATION

To date the US Government response to this new antivaccine reality has been modest although not insubstantial. The US Surgeon General, Dr Vivek Murthy, recently issued advisories about online disinformation and alerted the nation to its dangers,^34^ while the Department of Health and Human Services currently works with the tech giants to alter computer algorithms in order to limit its dissemination and spread. In some cases, this approach reflects the complexities and public perceptions of going up against constitutional limits on free speech. While helpful, such efforts may not move the needle on immunizations unless a path is identified to halt those who generate the antivaccine and antiscience content. Now, an added concern is the global impact. American-style antivaccine activism has spread to the world’s low- and middle-income countries, while, in parallel, authoritarian governments and leaders have adopted similar antivaccine rhetoric and actions.^33^

A reality has set in: the health sector overall appears paralyzed or at least confused by the fact that antiscience has morphed into a dominating political enterprise. This reality is also deadly, killing more Americans, as well as others in Europe, Latin America, and elsewhere, than other forms of political terrorism. And yet, we do not frame it as such. While everyone is entitled to their political views, even extreme ones, we must find a way to uncouple them from deadly antiscience propaganda. More and more, the antivaccine framework is now heavily imbued with the imagery of Nazi era atrocities and relies on discrediting, humiliating, or threatening scientists and physicians, including many who are Jewish. Antiscience has become an opportunity to openly and brazenly express anti-Semitic tropes and beliefs. Beyond the obvious humanitarian concerns of this new reality is the potential for it to unravel the modern structure of biomedical science, and cause our health and global security to suffer.

Turning back the tide on this new political face of antivaccine and antiscience aggression, or its expanded links to anti-Semitism, is not straightforward. Past approaches that focused on providing timely and accurate vaccine information remain essential, but increasingly they are inadequate to overcome this powerful new force now firmly embedded in the politics of the United States and authoritarian governments worldwide. The same is true for its anti-Semitic leanings. Because antivaccine activism is now firmly embedded in national political infrastructures and has moved beyond the health sector, we must recognize how traditional public health approaches will not be adequate for a counter response. Therefore, we must look beyond the health sector to experts in modern socioeconomics and geopolitics. The stakes are high, given that antivaccine activism is rapidly expanding and taking on an increasingly dark and sinister element.

## Abbreviations

CPAC: Conservative Political Action Coalition
Hib: *Haemophilus influenzae* type b
PAC: political action committee
WHO: World Health Organization
